# Present Advances and Future Perspectives of Molecular Targeted Therapy for Osteosarcoma

**DOI:** 10.3390/ijms17040506

**Published:** 2016-04-06

**Authors:** Atik Badshah Shaikh, Fangfei Li, Min Li, Bing He, Xiaojuan He, Guofen Chen, Baosheng Guo, Defang Li, Feng Jiang, Lei Dang, Shaowei Zheng, Chao Liang, Jin Liu, Cheng Lu, Biao Liu, Jun Lu, Luyao Wang, Aiping Lu, Ge Zhang

**Affiliations:** 1 Institute for Advancing Translational Medicine in Bone & Joint Diseases, School of Chinese Medicine, Hong Kong Baptist University, Hong Kong 999077, China; aatikshaikh@gmail.com (A.B.S.); fayebalaba@live.com (F.L.); minlee123@163.com (M.L.); hebinghb@gmail.com (B.H.); hxj19@126.com (X.H.); borisguo@hkbu.edu.hk (B.G.); lidefang@163.com (D.L.); jiangfeng@nbu.edu.cn (F.J.); danglei_hkbu@163.com (L.D.); liangchao512@163.com (C.L.); liujin_hkbu@163.com (J.L.); lv_cheng0816@163.com (C.L.); 13261526031@163.com (B.L.); ljaaa111@163.com (J.L.); luyaoben@126.com (L.W.); 2 Department of Orthopaedic Surgery, Shenzhen Hospital, Southern Medical University, Shenzhen 518100, China; 3 Orthopaedic Surgery Department, Nanfang Hospital, Southern Medical University, Guangzhou 510515, China; chenguofen12@163.com; 4 Department of Orthopaedic Surgery, the First Hospital of Huizhou, Huizhou 516000, China; zswljq@126.com

**Keywords:** osteosarcoma, molecular targets, targeted therapy, molecular mechanism

## Abstract

Osteosarcoma (OS) is a bone cancer mostly occurring in pediatric population. Current treatment regime of surgery and intensive chemotherapy could cure about 60%–75% patients with primary osteosarcoma, however only 15% to 30% can be cured when pulmonary metastasis or relapse has taken place. Hence, novel precise OS-targeting therapies are being developed with the hope of addressing this issue. This review summarizes the current development of molecular mechanisms and targets for osteosarcoma. Therapies that target these mechanisms with updated information on clinical trials are also reviewed. Meanwhile, we further discuss novel therapeutic targets and OS-targeting drug delivery systems. In conclusion, a full insight in OS pathogenesis and OS-targeting strategies would help us explore novel targeted therapies for metastatic osteosarcoma.

## 1. Introduction

**Figure 1:**
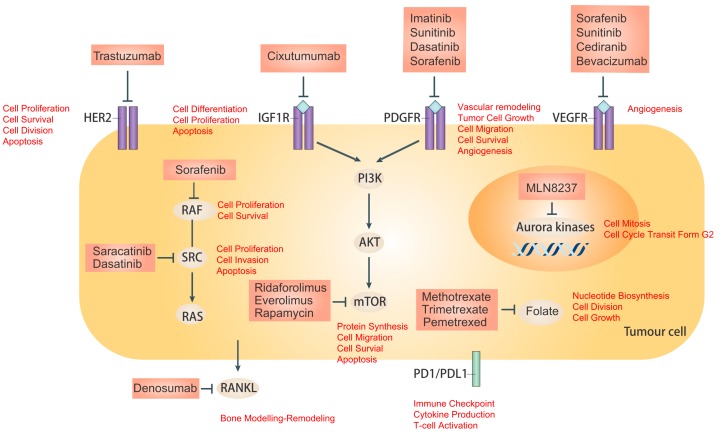
Overview of the targeted therapies in Osteosarcoma. This figure schematically shows molecular targets and associated drugs identified for therapeutic intervention in osteosarcoma. Therapeutic targets include specific cell surface receptor tyrosine kinases (RTKs): HER2, insulin-like growth factor 1 receptor (IGF1R), platelet-derived growth factor receptor (PDGFR), vascular endothelial growth factor receptor (VEGFR); and intracellular signaling targets: steroid receptor co-activator (SRC), RAF, mTOR and aurora kinases. Other potential targets include receptor activator of nuclear factor-κB ligand (RANKL), AKT, mTOR, programmed cell death 1 (PD-1)/programmed cell death ligand 1 (PD-L1), and folate.

Osteosarcoma (OS) is a primary bone cancer, predominantly affecting children and adolescence population [1]. OS originates from primitive mesenchymal bone forming cells and often occurs in long bones, such as proximal tibia and distal femur [2,3]. Current OS treatment regime consists of the combination of surgery and intensive multi-agent chemotherapy. Ever since the introduction of chemotherapy, five-year survival rate among OS patients has improved to 60%–75% [4]. However, 30%–40% of OS patients are associated with pulmonary metastasis and relapse, which have significantly poor prognosis, with an overall five-year survival rate of about 20% [5]. Moreover, over the past few decades, no substantial improvement in survival rate has been achieved, though efforts were made by intensifying dosing, varying timing and using multi-combinational chemotherapy. Additionally, several adverse effects are accompanied by high-dose chemotherapy [6]. Hence, there is an increasing sense of urgency to identify new biological markers and develop novel, innovative and specific molecular targeted therapeutic approaches to improve the outcome in osteosarcoma patients with poor prognosis. In this review, we will discuss about molecular mechanism underlying osteosarcoma, current molecular therapeutic targets against immune system, extracellular and Intercellular signaling transduction pathway of the bone metabolism, as well as novel therapeutic targets and drug delivery systems that have been investigated or are currently undergoing investigation in translational studies (Figure 1).

Molecular mechanism underlying osteosarcoma is characterized by complex karyotype, extensively unstable genome and complicated interaction between multiple proteins and signaling pathways [7,8]. Several groups have reported association between mutations and tumor suppressor genes. Retinoblastoma 1 (*RB1*) and tumor protein *p53* (*TP53*) have been implicated in osteosarcoma pathogenesis. Mutations in the *Rb1* gene and high frequency of allelic loss of *Rb1* gene at 13q and *p53* gene at 17p have been detected in OS samples [9,10]. Moreover *RB1* inactivation was detected in almost 20%–40% of OS cases and associated with poor prognosis [11]. Inactivation or loss of *TP53* causes cells to be resistant to DNA damage as well as loss control of cell growth thus resulting in extensive proliferation, transformation, and evade toxic effects of DNA damaging agents [12]. Recent studies have identified, several genes including Apurinic/Apyrimidinic exonuclease 1 (*APEX1*), *Myc* and epidermal growth factor receptor 2 (*HER2/neu*, *ErbB2*) in osteosarcoma pathogenesis. *APEX1* gene were found to be amplified with their respective protein overexpressed and could also correlate well with recurrence, metastasis, and survival in osteosarcoma patients [13]. *Myc* is a transcription factor that stimulates cell growth and mitosis. High expression of *Myc* in bone marrow stromal cells caused loss of adipogenesis and transformation into osteosarcoma [14]. *Myc* was also found to be amplified in OS cells lines resistant to conventional chemotherapy [15]. Higher levels of human epidermal growth factor receptor 2 (*HER2/neu*, *ErbB2*) in OS patient samples were demonstrated to be associated with early pulmonary metastases and poor prognosis [16]. In addition, other molecules which have been found to be associated with osteosarcoma pathogenesis are bone morphogenetic protein type II receptor (BMPR2) and high mobility group box1 (HMGB1) [17,18]. OS tumor pathogenesis is not bound to a specific gene mutation or gene loss or to aberrant signaling pathways, but a complex entangled mechanism. Therefore, several molecules are being investigated in order to develop targeted therapies for OS.

## 2. Immunomodulators

### 2.1. Interferons

Interferons (IFNs) are a group of signaling proteins known as cytokines, which stimulate immune system and induce anti-angiogenic and antitumor activity. IFNs have successfully demonstrated high clinical activity associated with several types of cancers [19]. IFN-α-2b has shown to exhibit inhibition on the growth of patient-derived OS cells and also on the tumor growth in PDX mouse models. In a pilot study of non-metastatic OS patients treated with IFN-α for 3–5 years, five-year disease-free survival at 63% was reported [20]. Additionally, high-grade osteosarcoma patients treated with IFN-α for 3–5 years achieved 10-year disease-free survival at 43% [21]. A recent report investigated the efficacy of IFN-α-2b as a maintenance therapy in OS patients with good response to MAP (methotrexate, doxorubicin, and cisplatin) induction chemotherapy, but no improvement in outcomes with event-free survival (EFS) were presented [22].

### 2.2. GM-CSF

Granulocyte-macrophage colony-stimulating factor (GM-CSF) is also an immunomodulatory cytokine tested in osteosarcoma. As an effective immunomodulator, the activity of GM-CSF against melanoma and Ewing sarcoma has been well demonstrated [23,24]. A phase I study of aerosolized GM-CSF showed that it was well-tolerated and effective in selected melanoma and Ewing sarcoma patients [25]. Moreover, a phase II trial of aerosolized GM-CSF on OS patients with first recurrence of pulmonary metastasis demonstrated that it was a feasible and safe treatment option. However, GM-CSF had no signs of immunostimulatory effects on pulmonary metastases or any improvement in outcome in OS patients [26].

### 2.3. Mifamurtide

With immune stimulatory activity, muramyl dipeptide (MDP) is the minimal bioactive peptidoglycan component in the cell wall of Bacille Calmette–Guerin mycobacterium [27]. Mifamurtide (MTP-PE or muramyl tripeptide phosphatidyl ethanolamine) is a synthetic derivative of MDP. Liposome encapsulation of mifamurtide favors targeted delivery of MTP-PE to monocytes and macrophages, which activates the tumoricidal activity of macrophages and monocytes via secreting interleukin(IL)-6, tumor necrosis factor (TNF)-αand increased phagocytosis[28,29]. Early *in vivo* activity of mifamurtide was reported in dogs with spontaneous OS, and the treatment with MTP-PE following amputation had significantly improved disease-free survival to 222 days, compared to 77 days in the placebo group [30,31]. Since then, several clinical trials have been performed in humans. A Phase III, randomized, prospective intergroup trial (INT-0133) study of mifamurtide on patients with newly diagnosed osteosarcoma, showed significant improvement in six-year overall survival from 70% to 78% and in patients with metastatic disease showed improvement in five-year overall survival from 40% to 53% [32,33]. Numerous studies have reported of promising clinical benefits when mifamurtide is combined with chemotherapy in treatment of metastatic OS [34]. The drug has been currently approved as an adjuvant treatment of osteosarcoma by European Medical Agency, but has not been approved by the US FDA. Hence, given the promising data, further investigation is needed to clarify the role of mifamurtide in treatment of OS. Currently, several clinical trials of mifamurtide’s effectiveness in treating OS are being conducted.

## 3. Tyrosine Kinase Receptor Inhibitors

### 3.1. Receptor Tyrosine Kinases (RTKs)

RTKs are cell-surface receptors which play a key role in the activation of multiple downstream signaling pathways including, phosphatidylinositol 3 (PI3)/Akt kinase and extracellular signal regulated kinase (Erk) [35]. And hence is an important mediator in regulation of normal cellular as well as physiological processes such as cell growth, survival and proliferation. Moreover, RTKs have been arraigned as a key factor in growth and progression of several tumors and several gene mutation, amplification have been implicated in the disruption of RTKs signaling cascade [36]. Here we list a few RTKs undergoing clinical trials that are involved in pathogenesis of OS (Table 1).

### 3.2. Insulin-Like Growth Factor Receptor (IGF-R)

IGF-R is a membrane tyrosine kinase receptor and presents in two subtypes IGF-1R and IGF-2R. IGF-1R is a transmembrane RTK and has been implicated in mediating cell differentiation, proliferation, and apoptosis in several cancers [48]. The insulin-like growth factor-1 and 2 (IGF-1 and IGF-2) bind to the IGF-1R, undergoes auto-phosphorylation and activates two major oncogenic signaling cascades: the phosphoinositide 3-kinase (PI3K/Akt) pathway and the mitogen-activated protein kinase (MAPK) pathway [49,50]. IGF-R overexpression has been observed in several OS cell lines, and is reliant on activation of IGF-1R by IGF- 1 to stimulate OS cell growth and metastatic dormancy in *in vivo* and *in vitro* [48,51]. Also IGF-R levels were seen to be elevated among OS patients tumor samples and further the elevated expression of IGF-1R and IGF-1 ligand correlated with the poor prognosis and survival rate in OS patients [52,53].

Current anti-IGF-R therapeutic approaches consist of human monoclonal antibodies (mAbs) targeting IGF-1R, IGF ligand-neutralizing antibodies and small-molecule tyrosine kinase inhibitors of IGF-1R. Several human monoclonal antibodies (mAbs) targeting IGF-1R has been developed and some of them has been or are being investigated in different clinical trials. Cixutumumab is a fully human IgG1 mAbs specifically targeting IGF-R. Phase I/II clinical trial of cixutumumab on children with refractory solid tumors including OS, reported cixutumumab to be well tolerated but with limited single-agent activity [37,38]. Initial phase II trials, combination of cixutumumab and the mTOR inhibitor temsirolimus had shown clinical activity, but a recent phase II trial could not achieve the objective response. Studies on another fully human mAb SCH 717454 (robatumumab), had revealed it to be less effective *in vitro* but had significant tumor regression by inhibiting cell proliferation and angiogenesis in several OS xenograft models [54]. Furthermore, SCH 717454 in combination with cisplatin or cyclophosphamide had demonstrated a remarkable increase in *in vivo* antitumor activity compared with single agent treatment [54]. However, a phase 1/1B trial of SCH 717454 in combination with different treatment regimens in pediatric patients with advanced solid tumors (NCT00960063 *) and a phase II trial on activity of SCH 717454 in patients with relapsed OS and Ewing’s sarcoma (NCT00617890 *) were recently terminated (Table 1).

Two IGF ligand-neutralizing antibodies against IGF ligands IGF-I and -II have been found: BI836845 and MEDI-573. Both BI 836845 and MEDI-573 are fully human monoclonal antibody with high affinity towards IGF ligands IGF-I and IGF-II, and selectively neutralizes the bioactivity of IGF ligands thereby down regulating IGF signaling through both the IGF-1R and IR-A pathways [55,56]. At present, several phase I trials of BI836845 for patients with solid tumors are ongoing recruitment (Table 1). In addition to mAbs, small-molecule tyrosine kinase inhibitors of IGF-1R are also being investigated. BMS-754807 is a reversible small molecule ATP-competitive inhibitor of IGF-1R. Evaluation of BMS-754807 against the Pediatric Preclinical Testing Program (PPTP) has reported to have a broad activity towards pediatric sarcomas and xenograft models through IGF-1R inhibition and efficacy of BMS-754807 could be enhanced when used in combinations, by targeting multiple, related signaling pathways [57]. However, even though anti-IGF-1R therapeutic approach has shown clinical benefits in some pediatric subjects, several clinical trials on anti-IGF-1R agents have been terminated, because the observed clinical benefits of targeting IGF-1R pathway in single and multi-agent strategies could not met the primary response.

### 3.3. Platelet-Derived Growth Factor/Receptors (PDGF/PDGFR)

Platelet-Derived Growth Factor/receptors (PDGF/PDGFR) are signaling molecules which play an important role in cell proliferation and differentiation of osteoblasts and osteoclasts [58]. PDGF/PDGFR signaling pathway, more specifically PDGFR-α/PDGF-A has been implicated in tumor growth and metastasis in variety of human solid tumors, including OS and the overexpression of PDGFR correlates with the disease progression and poor prognosis [59,60]. While some conflicting reports disagree about correlation between PDGF-A expression and prognosis in OS [61]. A recent *in vivo* study on OS cells–platelet interactions reported that OS cells, when in contact with platelets, could induce platelet aggregation and stimulate release of platelet-derived growth factor (PDGF) from platelets. Furthermore, platelets cause phosphorylation of PDGFR and Akt, there by promoting the proliferation of OS cells [62].

Imatinib mesylate (STI-571) is potent tyrosine kinase inhibitors against PDGFR signaling and was originally developed for the treatment of chronic myelogenous leukemia (CML). Imatinib mesylate also has multi-targeted inhibitory activity towards several kinases including, c-KIT and Bcr/Abl [63]. *In vitro* studies of imatinib mesylate on various OS cell lines had demonstrated imatinib mesylate to have variable toxicity and could not be viewed as a single agent for the treatment of osteosarcoma [64], while another study had reported imatinib mesylate to exert a dose-dependent anti-proliferative effect in most of the human, mouse and rat OS cell lines through cell cycle arrest, inducing caspase-dependent cell death and inhibiting cell migration. Additionally, in murine syngeneic models of undifferentiated- and mixed osteoblastic/osteolytic forms of OS, imatinib mesylate could inhibit the tumor growth in both preventive and curative approaches [65]. However, in a phase II clinical trial of imatinib mesylate in children with refractory or relapsed solid tumors including OS, it failed to demonstrate significant response [42] (Table 1).

Other multi-targeted RTK inhibitors, which also block PDGF/PDGFR signaling, are sunitinib and dasatinib. Sunitinib is a small molecule, which is approved for treatment of renal cell carcinoma (RCC) and imatinib-resistant gastrointestinal stromal tumor (GIST). Sunitinib can also inhibit VEGFR and FLT-3 (fms-related tyrosine kinase-3). Sunitinib showed anti-tumor activity in *in vitro* as well as in OS xenograft models, but this may be due to anti-angiogenic effect instead of PDGFR inhibition [66,67]. In a phase 1 trial of sunitinib in pediatric population with solid tumors, initially treated with anthracyclins, reported best response in four patients including one OS patient, with toxicities mainly representing hematological and cardiac symptoms [43,68] (Table 1). Dasatinib a multi-targeted RTK inhibitors also inhibits SRC and BCR-ABL [69].

### 3.4. Vascular Endothelial Growth Factor (VEGF)

The vascular endothelial growth factor (VEGF) ligands and receptors (VEGFR) are implicated in tumor angiogenesis and play an important part in tumor growth and development. VEGF receptor consists of a family of receptors (VEGFR-1, VEGFR-2 and VEGFR-3) which could be activated by their ligands the VEGFs (A, B, C and D) [70]. VEGF overexpression has been observed in several tumors including OS. In particular, expression levels of VEGFR-3 correlated with pulmonary metastasis development and overall survival in patients with OS [71,72]. Several preclinical studies have revealed that specifically targeting VEGFs ligands or its receptors, may be an effective strategy in treating OS [73].

At present VEGF-based anti-angiogenic therapies, include anti-VEGF antibodies and small-molecule tyrosine kinase inhibitors against VEGFR. Bevacizumab, is a humanized monoclonal antibody (mAb) targeted towards the VEGF receptor and had shown some beneficial preclinical results [74]. However, phase I evaluation of bevacizumab in children with refractory solid tumors including OS, reported no observable response [44]. Currently a phase I and II trail of bevacizumab in combination with several chemotherapy agents on OS is ongoing (NCT00458731 *) (Table 1).

The PPTP has successfully identified three potent small molecule TKIs, which selectively target VEGF receptor family. These are sunitinib, sorafenib and cediranib. Sunitinib and sorafenib are multi- targeted inhibitors, with overlapping targets (PDGFR, FLT3, RET, KIT and VEGFR). Sorafenib was first approved for the treatment of un-resectable hepatocellular carcinoma (HCC). Preclinical studies of sorafenib, reported promising results with reduction in tumor growth and inhibit pulmonary metastasis in OS models [75,76]. In a phase II study of sorafenib as single treatment agent in patients with relapsed and unresectable OS, progression-free survival (PFS) at four months was 46%, while overall survival was seven months, objective response was seen in 14% of patients and 29% of patients had stable disease [45]. Further clinical evaluation of sorafenib in combination with bevacizumab (NCT00665990) and irinotecan (NCT01518413) are in progress (Table 1).

### 3.5. Human Epidermal Growth Factor Receptor 2 (HER-2)

HER-2 is RTKs and belongs to the human epidermal growth factor receptor (HER/EGFR/ERBB) family. HER-2 expression is linked to the development and progression of several tumors. Recent development of targeted therapies directed towards HER-2 have achieved significant benefits in the treatment of several solid tumors [77]. However, there are some conflicting reports regarding HER-2 expression in OS. While, some studies have reported over expression of HER-2 in 40% of OS patient samples and its increased expression correlated with metastasis, relapse and poor prognosis [78,79]. However, other studies have reported minimal HER-2 expression in OS tumor samples or no association between cytoplasmic HER-2 expression in OS tumor samples and patient’s prognosis [80,81].

Trastuzumab is a monoclonal antibody targeting HER-2 receptors and was primarily used in the treatment of breast cancer. In a phase II study of trastuzumab in addition to conventional chemotherapy has been shown to be safe, but with no clinical benefits. Briefly, this study also demonstrated non-significant difference in event-free and overall survival between the HER-2-positive group treated with trastuzumab and the HER-2 negative group treated with conventional chemotherapy [46,82] (Table 1).

## 4. Intracellular Signaling Inhibitors

### 4.1. Steroid Receptor Co-Activator (Src)

Src is non-receptor protein TK. Src is an essential molecule required for normal osteoclast activity. However, over expression and activation of Src is also well associated with cancerous cell survival, development, adhesion and invasion, through various downstream signaling pathways such as Ras/mitogen-activated protein kinase pathway, STAT-3, FAK and c-Jun kinase. There by resulting in an overall aggressive and metastatic potential. High level of Src have been expressed in variety of cancers including OS [83]. Inhibition of Scr Phosphorylation *in vitro* induces pro-apoptotic activity and decreases cell invasion, migration, and adhesion. However, similar results could not be replicated using OS *in vivo* models, suggesting that Src activation may not be the primary pathway of pulmonary metastases in OS [84,85,86]. Dasatinib is a small-molecule tyrosine kinase inhibitor, directed against Src. Dasatinib is presently used in the treatment of chronic myeloid leukemia (CML) and Acute lymphoblastic leukemia (ALL). A clinical trial on pharmacokinetics and drug interaction of dasatinib showed no complete or partial responses, but maintained a stable disease condition in OS patient [8,87,88]. Two clinical trials to investigate either dasatinib alone or in combination are ongoing and awaiting results (Table 2). Saracatinib is a dual-specific inhibitor of Src and Abl. A few phase I trials of saracatinibin solid tumors have been conducted, but no OS patients were included in those studies [89,90]. Currently, Phase II studies of Src inhibitor saracatinibin recurrent pulmonary metastatic OS are being conducted (Table 2).

### 4.2. The Mammalian Target of Rapamycin (mTOR)

mTOR is a serine/threonine protein kinase, involved in PI3K/Akt signaling pathway and is responsible for regulation of protein synthesis, cell cycle and cell survival [96]. Activation of mTOR is attributed to be one of the key mechanisms in development and progression of several cancers including OS. Moreover, high level of mTOR in OS patients have been consistently correlated with poorer prognosis [97,98]. Indeed, suggesting mTOR as a potential therapeutic target.

The first generation of mTOR inhibitors are rapamycin (sirolimus) and rapamycin analogs like everolimus, temsirolimus, and ridaforolimus, which inhibit the kinase activityof mTORC1 through binding to FKBP-12 [99]. Preclinical testing of rapamycin monotherapy, demonstrated broad antitumor activity in *in vivo* as well as in OS xenograft models. Moreover, combination of rapamycin with cyclophosphamide or vincristine, enhanced the response in the OS models [100,101]. However, a clinical trial of sirolimus and cyclophosphamide in OS patients did not have a satisfactory response [91]. Clinical studies of everolimus in pediatric patients with refractory solid tumors (including 2 OS patient) had reported everolimus to be safe and had pharmacokinetic properties to those observed in adults, with stable disease observed in a single OS patient [92]. While the combination therapy of mTOR inhibitor everolimus and IGF-1R inhibitor figitumumab had no positive response in OS patients [93]. A non-randomized phase II trial on combination of sorafenib and everolimus in high-grade non-resectable osteosarcoma patients reported favorable antitumor activity as a 2nd or 3rd line of treatment, but could not attain the target of 6 months progression free survival in 50% patients [45]. Temsirolimus an analog of rapamycin, when used in combination with cisplatin or bevacizumab has demonstrated superior antitumor activity in comparison to their respective monotherapies in OS models [102]. Furthermore, in clinical setting, combination of temsirolimus with cixutumumab has shown positive clinical activity in patients with bone and soft-tissue sarcoma [40]. In a phase II trial of ridaforolimus, a selective mTOR inhibitor as a single agent therapy in patients with advance sarcoma, shows partial response in 2 OS patients (2/4 patients with osteosarcoma), while a larger double-blind phase III study has shown an increase in progression free survival (PFS) in patients with metastatic sarcoma after effective prior chemotherapy (50 bone sarcoma patients). However the study does not provide subset analysis for the bone sarcoma patients [103,104].

The second generation of dual mTOR inhibitors like, AZD8055, which inhibit mTOR1 as well as mTOR2, has demonstrated weak anti-tumor activity in OS xenograft. While, the effectiveness of other novel small-molecule inhibitors such as dual PI3K/mTOR inhibitors (NVP-BEZ235) and dual mTOR/DNA-PK inhibitor (CC-115) are still under clinical investigation [105,106]. Currently, several clinical trials of different types of mTOR inhibitors in treatment of OS are ongoing, either as a single agent therapy or as combinational therapy with other targeted agents (Table 2). These studies might give a better insight into the effectiveness of mTOR inhibitors in OS treatment.

### 4.3. Aurora Kinase

Loss of cell cycle control is a hallmark characteristic of cancer cells, thus leading to uncontrolled cell proliferation. Aurora kinase are serine/threonine protein kinases that regulate the normal cell mitosis, cell cycle transit from G2 and are potential oncogenic activators, as their abrupt expression or activation could cause normal cells to transform into cancerous cells [107]. In mammalian cells the aurora kinase gene encodes the three aurora kinase proteins: AURK-A, AURK-B, and AURK-C. Among these three types, AURK-A has been mostly involved in the tumorigenesis of several cancers including OS. Preclinical studies on silencing of AURK-A protein expression in osteosarcoma cells revealed that it could induce cell cycle arrest in G2/M and result in cell apoptosis *in vitro* [108,109]. Many aurora kinase inhibitors have been found and are being investigated in different stages of drug development.

MLN8237 (alisertib) is a second generation aurora kinase inhibitors, specifically targeting AURK-A via competing with ATP binding. Initial testing of MLN8237’s effectiveness, showed complete responses in one of six OS xenografts models [110]. A recent *in vitro* study has reported that MLN8237 through aurora kinase inhibition could induce autophagy and apoptotic cell death via PI3K/Akt/mTOR, p38 MAPK, and AMPK signaling pathway and that the reactive oxygen species (ROS) as well as sirtuin-1 pathways are also involved [111]. MLN8237 has been inducted in several clinical trials some of which are ongoing or awaiting results (Table 2).

## 5. Future Perspectives

### 5.1. Rank Inhibitors

OS is strongly associated with the receptor activator of nuclear factor-κB (RANK), along with its ligand (RANKL) and decoy osteoprotegerin (OPG). The molecular triad of RANK, RANKL and OPG play a key role in normal homeostasis between bone formation and bone loss, during bone remodeling. An imbalance in this triad has been associated with pathological bone remodeling and metabolism. Hence, the cancerous cells capitalize on the molecular triad of RANK, RANKL and OPG, thereby causing osteolytic or osteoblastic bone metastases [112,113]. An increased expression of RANK in human OS cell lines have been reported and the RANK activation further resisted anchorage-independent cell death [114]. However, inhibiting RANK expression in OS cells with shRNA, had reduced cell invasion and motility, while had limited effect on cell proliferation [115]. Additionally, RANKL has high expression in OS patients. A retrospective study reported two-thirds of OS biopsy specimens had positive staining for RANKL and this is poor prognosis in OS patients with high RANKL expression [116]. Furthermore, an *in vivo* study had demonstrated that the decoy osteoprotegerin (OPG) could indirectly prevent tumor-induced osteolysis and also inhibit tumor growth [117]. Hence, the molecular triad of RANK, RANKL and OPG could be a valuable therapeutic target in treating RANK-positive osteosarcoma.

Denosumab is a recombinant, humanized IgG 2 monoclonal antibody that is specifically targeted towards RANKL. Denosumab was initially introduced for treatment of osteoporosis as it prevents bone resorption by inhibiting differentiation of osteoclast precursors into mature osteoclasts. And it was later found to be beneficial in treatment of patients with skeletal-related events (SREs) from breast cancer and giant cell tumors of bone [118,119]. A phase II trial of denosumab has shown rapid and sustained effects in patients with giant cell tumor of bone. A randomized, double-blind study, presented superior efficacy of denosumab to zoledronic acid in delaying or preventing SREs in patients with breast cancer metastatic to the bones [120]. Given these results, further investigation in development of denosumab as treatment choice for patients suffering from high expression of RANKL in OS may be warranted.

### 5.2. Programmed Cell Death 1 (PD-1)/Programmed Cell Death Ligand 1 (PD-L1) Pathway

Recently, there has been interest in better understanding of cellular immune mechanism, especially about PD-1 and its ligand (PD-L1). PD-1 is a transmembrane protein, which is expressed on immune cells, activated T cells, B cells, natural killer (NK) T cells, activated monocytes, and dendritic cells. PD-1, functions as an immune checkpoint, by activation of PD-1/PD-L1 signaling pathway, promotes suppression of autoimmunity by preventing T-cells activation and by reducing cytokine production [121]. Over expression of PD-1 was found in several human cancers and its expression is associated with the disease progression as well as poor prognosis [122]. A recent report, revealed that programmed cell death ligand 1 (PD-L1) were highly expressed in OS patients, while another study reported that human metastatic tumors and not primary, OS tumors express PD-L1. This study indicated that blockade of PD-1/PD-L1 signaling pathway could drastically improve the functions of tumor-infiltrating lymphocytes, reduce the tumor burden and increase survival in OS mouse models [123]. Hence, based on supporting clinical evidence, targeting PD-1/PD-L1 interactions by anti-PD-1 antibody or PD-1 inhibitors may be a potential therapeutic strategy for the treatment of OS.

### 5.3. MicroRNAs

MicroRNAs, a class of 22-nucleotide small noncoding RNAs, are crucial for regulation of several pathophysiological processes and have been found to be involved in tumorigenesis, tumor progression and metastasis. It was found that miR-144 expression was significantly lower, while ROCK1 and ROCK2 levels were elevated in OS cell lines and OS tissues, further studies on restoration of miR-144 expression resulted in significant reduced cell proliferation, migration, invasion suppressed metastasis *in vivo* and *in vitro*, suggesting that miR-144 is a potent tumor suppressor by down regulating ROCK1 and ROCK1 expression in OS [124,125]. Thus, restoring miR-144 expression could be beneficial in treating osteosarcoma. Similarly, introduction of miR-132 suppressed OS cell proliferation *in vitro*, as well as in OS xenograft via cyclin E1 (CCNE1), while another study showed that miR-132 suppressed OS cell malignant behavior via Sox4 [126,127]. Elevated levels of miR-27a and overexpression of miR-27a/miR-27a pair have shown to suppress CBFA2T3 expression and thus contributes to development of pulmonary metastasis in OS [128,129]. MicroRNAs are also associated with the chemo resistance in OS. High expression of miR33a was found in OS cells that are resistant to cisplatin, through down regulation of TWIST and that inhibition of miR33a could increase the cell apoptosis induced by cisplatin [130] Therefore, microRNAs like miR-144, miR132, miR-27a, miR33a and may be able to serve as a diagnostic and therapeutic target for OS treatment.

### 5.4. Anti-Folate

Initially, high-dose methotrexate was considered as a standard treatment for osteosarcoma, but there is an increase in the number of cases of methotrexate resistant OS due to loss or reduced function of the reduced folate carrier protein and less retention of methotrexate within the cell, which led to the discovery of anti-folate agents like trimetrexate and pemetrexed. Trimetrexate is a better alternative to methotrexate for patients with osteosarcoma, as it does not rely on reduced folate carrier to enter into the cells. Also higher impaired methotrexate transport was found in OS patients samples, which is thought to have been caused due to defect in functionality of the reduced folate carrier protein [131]. Trimetrexate has demonstrated positive response in OS. A clinical study showed one complete response and one partial response in trimetrexate treated OS patients (seven evaluable patients) [132]. Pemetrexed is a multi-targeted anti-folate that inhibits several functional folate-dependent enzymes and DNA synthesis including thymidylate synthase (TS), dihydrofolate reductase and glycinamide ribonucleotide formyl transferase (GARFT) and is also dependent on reduced-folate carrier to be transported into the cells [133,134]. Pemetrexed has shown significant anti-tumor activity in OS cell lines but not superior to methotrexate [135]. In clinical setting, pemetrexed as a second line therapy in patients with advanced/metastatic OS has demonstrate partial response in one and stable disease in five patients (total 32 patients) suggesting insufficient minimal response [94]. Similar results were reported in another phase II study of pemetrexed in pediatric patients with refractory solid tumors including OS, with stable disease in one OS patient, with overall no observable response [95]. Different anti folate agents are still being tested in clinical trials (Table 2).

### 5.5. Improved Delivery Systems

A new therapeutic strategy in treating pulmonary metastasis in OS is to modify the delivery system of current chemotherapy agents so as to enhance local efficacy of chemotherapy agents. One such strategy is by liposomal encapsulation of drug and aerosolized delivery. Initial phase Ib/IIa study of the sustained release lipid inhalation targeting (SLIT) cisplatin in patients with OS metastatic to the lung has reported to be well tolerated and had pulmonary disease free in two patients (2/14 patients) at one year after start of therapy [136]. Further clinical study on efficacy of inhaled lipid cisplatin (ILC) in recurrent/progressive metastatic OS patients demonstrated partial response in one and stable disease in two patients (19 patients under evaluation) [137]. A phase II trial of ILC in patients with recurrent pulmonary OS is ongoing. Doxorubicin is one of the most active first-line chemotherapy agents used in the treatment of OS patients. Preclinical studies of liposomal encapsulation of doxorubicin has shown significant anti-tumor activity *in vitro* and *in vivo* [138]. Furthermore, a phase I/II clinical study of liposomal encapsulated doxorubicin have shown to be active against doxorubicin sensitive tumors as well as doxorubicin resistant tumor types [139]. The use of an aerosolized version of IL-2 could cause site-specific immunomodulation in OS patients with pulmonary malignancies and thus reduce drug-induced toxicities caused by the systemic administration. Phase I and II clinical trials (NCT01590069 *) are presently ongoing and the results of these studies will provide deep insights into the clinical benefits of these therapeutic approaches.

#### 5.5.1. Doxorubicin Conjugate

Efforts are being made to tailor-fit active first-line chemotherapy agents, to improve their efficacy in treating drug resistant OS. Aldoxorubicin (INNO-206) a prodrug of doxorubicin (formerly known as DOXO-EMCH) has surfaced as a potential therapeutic agent against doxorubicin resistant tumors. INNO-206 is a (6-maleimidocaproyl) hydrazone derivative of doxorubicin. It is an albumin-binding prodrug with doxorubicin attached to an acid-sensitive linker (EMCH). Mechanistically, aldoxorubicin binds covalently to serum albumin, which predominantly accumulates in and around the cancerous cells. The acidic environment at tumor site causes cleavage of the acid-sensitive linker and release of free doxorubicin. A series of phase I studies on DOXO-EMCH showed it to be safe, stable in circulation without accumulating in body compartments with fewer cases of doxorubicin-associated cardiotoxicity and could induce tumor regressions in tumor types known to be anthracycline-sensitive tumors such as breast cancer, small cell lung cancer and sarcomas [140,141,142]. Aldoxorubicin is presently under investigation in different phases of clinical trial (NCT02235688 *). However, clinical efficacy of aldoxorubicin in treating metastatic OS patients has yet to be explored.

#### 5.5.2. Nanoparticle Albumin-Bound (Nab) Paclitaxel

Paclitaxel (Taxol and Onxal) is cytotoxic microtubule stabilizing agent, against broad range of malignancies, including ovarian cancer, breast cancer, bladder cancer, head and neck cancer. However, its application in treating pediatric cancer patients has been limited due to its very low aqueous solubility, which requires paclitaxel to be formulated with Cremophor EL/ethanol as the solvent, which causes severe allergic, hypersensitivity and anaphylactic reactions. Nab-paclitaxel (Abraxane) is a novel, solvent-free, nanoparticle albumin-bound (nab) paclitaxel modified to reduce the toxicity of solvent-based paclitaxel. Nab-paclitaxel showed significant antitumor activity against most pediatric solid tumor cell lines including OS, while in OS xenograft model, nab-paclitaxel demonstrated significantly higher tumor inhibition (98.8%) compared to adriamycin (46.1%) and paclitaxel (40.8%). Further supporting the biologic rationale for clinical studies, nab-paclitaxel has shown to overcome paclitaxel resistance, mediated through ABCB1 observed in human OS cell lines [143,144,145]. Globally, nab-paclitaxel is approved for the treatment of metastatic breast cancer (MBC). At present, phase I/II clinical studies of nab-paclitaxel’s activity in pediatric patients with recurrent/refractory solid tumors including OS, are in the recruiting stage (NCT01962103 *).

## 6. Conclusions

Osteosarcoma is a highly heterogeneous bone cancer, which predominantly affect children and adolescence population. Current OS treatment regime consists of methotrexate, doxorubicin, cisplatin and ifosfamide, whose clinical benefits seem to have been maximized. Furthermore, strategies of intensifying chemotherapy dosing, varying its timing and introduction of multi-combinational chemotherapy has not helped to further improve the outcome of OS patients presenting with pulmonary metastasis and relapse. The identification of several new therapeutic agents, which specifically target immune system, cell proliferation, angiogenesis, extracellular and intercellular signaling pathways in tumor cells, either as single therapeutic agent or in combination with conventional chemotherapy has demonstrated promising data in preclinical and clinical trials. Indeed, a deeper insight into understanding genetic, molecular basis and tumorigenesis of OS, in addition to identification of biomarkers associated with poor prognosis and poor response to conventional treatment regimes, could pave the way to the discovery of novel molecular candidates that could be used in the development of potential therapeutic agents. In addition, the challenge ahead is to identify and harness these molecular therapeutic targets for multi-targeted combinational therapy or tailor-made personalized therapies or through modified drug delivery. This new approaches could help us explore the full potential of targeted therapies in OS.

## Figures and Tables

**Table 1:** Clinical trials of tyrosine kinase receptor inhibitors in osteosarcoma.

Target	Class	Therapy	Mechanism	Clinical Trial	Outcome	Reference
IGF-1R	Anti- IGF-R antibodies	Cixutumumab	Activation of (PI3K/Akt ) and MAPK	Phase I/II	PD	[37]
				Phase II	No OR	[38]
				Phase II	MPFS at 6 weeks	[39]
				Phase II	MPFS at 6 weeks	[40]
				Phase II	MPFS at 21.4 weeks	[41]
		Human mAb SCH 717454 (robatumumab)	Inhibits IGF-R binding and signaling	Phase I/IB (Terminated)	‒	NCT00960063 *
				Phase II (Terminated)	‒	NCT00617890 *
	IGF ligand-neutralizing antibodies	BI 836845 mAb	Neutralizes IGF ligand	Phase I	‒	NCT01403974 *
				Phase I	‒	NCT02145741 *
				Phase I	‒	NCT01317420 *
	Small-molecule TKI’s	BMS-754807	ATP-competitive inhibitor of IGF	Phase 1I	‒	NCT00898716 *
				Phase III	‒	NCT00134030 *
PDGFR	Small-molecule TKI’s	Imatinib mesylate	PDFGR, c-KIT	Phase II	no OR	[42]
				Phase II	‒	NCT00030667 *
				Phase II	‒	NCT00031915 *
	Multi-targeted RTK inhibitors	Sunitinib	PDGFR, FLT3, RET, KIT and VEGFR inhibitor	Phase I	SD in 1(2)	[43]
VEGF/VEGFR	Anti-VEGF antibodies	Bevacizumab	VEGF inhibitor	Phase I	no CPR	[44]
				Phase II		NCT00458731 *
	Small-molecule TKI’s	Sorafenib	PDGFR, FLT3, RET, c-KIT, VEGFR inhibitor	Phase II	45% PFS at 6 months	[45]
				Phase I	‒	NCT00665990 *
				Phase I	‒	NCT01518413 *
HER-2	Anti-HER-2 antibody	Trastuzumab	HER-2 inhibitor	Phase II	NSD	[46]
				Phase II	‒	NCT00005033 *

Abbreviations: PR, partial response; PD, progressive disease; SD, stable disease; MPFS, median progression-free survival; PFS, progression free survival; CPR, complete or partial response; OR, Objective response; NSD, no significant difference; * clinical trial number [47].

**Table 2:** Clinical trials of Intracellular signaling inhibitors in Osteosarcoma.

Target	Class	Therapy	Mechanism	Clinical Trial	Outcome	Reference
Src	small-molecule TKI’s (Multi targeted)	Dasatinib	PDGF/PDGFR, SRC, BCR-ABL inhibitor	Phase I	SD in 1(1)	[87]
				Phase II	SD in 5(45)	[88]
				Phase II	‒	NCT00464620 *
				Phase I/II	‒	NCT00788125 *
	Dual-inhibitor	Saracatinib	Src and Abl specific inhibitor	Phase II	‒	NCT00752206 *
mTOR	1st generation of mTOR inhibitors	Sirolimus	Inhibit mTORC1 by binding toFKBP-12	Phase II	No CPR	[91]
		Everolimus		Phase I	SD in 1(2), no OR	[92]
			‒	Phase I	SD in 3(3)	[93]
			‒	Phase II	‒	NCT01216826 *
		Temsirolimus	‒	Phase II	MPFS at 21.4 weeks	[41]
			‒	Phase II	MPFS at 6 weeks	[40]
			‒	Phase II	‒	NCT01614795 *
	2nd generation of dual mTOR inhibitors	AZD8055	mTOR1, mTOR2 inhibitor	Phase I	‒	NCT00731263 *
				Phase I withdrawn	‒	NCT01194193 *
Aurora kinase A	2nd generation aurora kinase inhibitors	MLN8237 (alisertib)	AURK-Ainhibitor via ATP binding	Phase I	‒	NCT02214147 *
				Phase I	‒	NCT01898078 *
				Phase II	‒	NCT01154816 *
				Phase I	‒	NCT02444884 *
Folate	Multitargeted antifolate	Pemetrexed	folate-dependent enzymes and DNA synthesis enzymes inhibitor	Phase II	CPR in 1	[94]
					SD in 5	
					PD in 22	
					MPFS at 1.4 months	
				Phase II	no CPR	[95]
				Phase II	‒	NCT00003776 *
				Phase II	‒	NCT00002738 *
				Phase I	‒	NCT00119301 *

Abbreviations: PR, partial response; PD, progressive disease; SD, stable disease; MPFS, median progression-free survival; PFS, progression free survival; CPR, complete or partial response; OR, Objective response; NSD, no significant difference;* clinical trial number [47].

## Abbreviations

OS	Osteosarcoma
SRC	Steroid receptor co-activator
PD-1	Programmed cell death 1
PD-L1	Programmed cell death ligand 1
RB1	Retinoblastoma 1
TP53	Tumor protein p53
APEX1	Apurinic/Apyrimidinic exonuclease 1
BMPR2	Bone morphogenetic protein type II receptor
HMGB1	High mobility group box1
RANKL	Receptor activator of nuclear factor-κB ligand
OPG	Osteoprotegerin
RTKs	Receptor tyrosine kinases
PDGFR	Platelet-derived growth factor receptor
VEGFR	Vascular endothelial growth factor receptor
IFNs	Interferons
MAP	Methotrexate, doxorubicin, and cisplatin
EFS	Event-free survival
GM-CSF	Granulocyte-macrophage colony-stimulating factor
MDP	Muramyl dipeptide
MTP-PE	Muramyl tripeptide phosphatidyl ethanolamine
IL	Interleukin
TNF	Tumor necrosis factor
PI3	Phosphatidylinositol 3
Erk	Extracellular signal regulated kinase
IGF-R	Insulin-Like Growth Factor Receptor
IGF	Insulin-Like Growth Factor
MAPK	Mitogen-activated protein kinase
PDGF/PDGFR	Platelet-Derived Growth Factor/receptors
RCC	Renal cell carcinoma
GIST	Gastrointestinal stromal tumor
FLT-3	Fms-related tyrosine kinase-3
HCC	Hepatocellular carcinoma
CML	Chronic myeloid leukemia
ALL	Acute lymphoblastic leukemia
AURK	Aurora Kinase
ROS	Reactive Oxygen Species
AMPK	5′ Adenosine monophosphate-activated protein kinase
RANK/RANKL	Receptor activator of nuclear factor-κB/Ligand
ROCK1	Rho-associated, coiled-coil-containing protein kinase 1
NK	Natural killer
nab	Nanoparticle albumin-bound
MBC	Metastatic breast cancer
TS	Thymidylate synthase
GARFT	Glycinamide ribonucleotide formyl transferase
